# Exploring the Experiences of Parents Whose Child has Received a Diagnosis of Autistic Spectrum Disorder in Adulthood

**DOI:** 10.1007/s10803-021-05296-y

**Published:** 2022-01-08

**Authors:** Hannah Legg, Anna Tickle, Alinda Gillott, Sarah Wilde

**Affiliations:** 1 Trent Doctorate in Clinical Psychology, Division of Psychiatry and Applied Psychology, University of Nottingham, YANG Fujia Building, B Floor, Jubilee Campus, Wollaton Road, Nottingham, NG8 1BB UK; 2 Neurodevelopmental Specialist Service, Nottinghamshire Healthcare NHS Foundation Trust, The Pines, Highbury Hospital, Bulwell, Nottingham, NG6 9DR UK; 3 Trent Doctorate in Clinical Psychology, Sarah Swift Building, University of Lincoln, Brayford Pool, Lincoln, LN6 7TS UK; 4 Present Address: The Ryegate Children’s Centre, Sheffield Children’s NHS Foundation Trust, Tapton Hill Rd, Sheffield, S10 5DP UK

**Keywords:** ASD, Adult diagnosis, Biographical illumination, Unmet needs, Thematic analysis

## Abstract

There is a growing trend of adult diagnosis of Autism Spectrum Disorder (ASD). Research has found that diagnosis can prompt a process of sense-making which may be disrupted by lack of post-diagnostic support. Given the continued involvement of many parents in supporting their adult son or daughter with ASD, it is vital to understand their experiences to meet their needs in adapting to the diagnosis. Eleven parents of recently diagnosed adults participated in semi-structured interviews which were analysed thematically. Findings demonstrate that the new knowledge of diagnosis facilitates changes in attributions, interactions and relationships, but can result in unmet emotional and relational support needs. Findings are relevant to those involved in adult diagnosis, and the provision of post-diagnostic support.

## Introduction

Autism Spectrum Disorder (ASD) is a lifelong neurodevelopmental condition characterised by impairments in social interaction and repetitive, restricted or stereotyped patterns of interests, activities and behaviours without significant delays in language or cognitive development (American Psychiatric Association, 2013; World Health Organisation, 2018). Proponents of the neurodiversity movement argue against the conceptualisation of ASD in terms of impairments, considering ASD a natural neurological variation (Ortega, 2009). They contend that it is the lack of accommodation in the social environment rather than any impairment that is disabling (Oliver, 1996), and that the value of people with ASD should be recognised and accepted by society (den Houting, 2019).

Although ASD is typically diagnosed in childhood, there is a trend of diagnosis-seeking in adulthood, possibly explained by growing public awareness (Hansen et al., 2015) and impairments becoming more apparent when faced with challenges of adult life (Young et al., 2011). When ASD is suspected in adults, the National Institute for Health and Care Excellence (NICE) guidelines (2016) recommend a comprehensive assessment. However, diagnosis of ASD in adulthood is challenging for reasons including lack of valid and reliable assessment measures (Wigham et al., 2019), service availability (Wigham et al., 2019), and social barriers such as anxiety, fear of not being believed, mistrust of healthcare professionals, difficulties describing symptoms, and stigma making it difficult for individuals to access assessment and diagnosis (Lewis, 2017).

Individuals diagnosed with ASD in adulthood experience complex reactions. Themes such as feelings of relief, anger, sadness and disappointment are common (Jones et al., 2014; Lewis, 2016; Powell & Acker, 2016; Punshon et al., 2009), as are improved self-understanding leading to increased self-acceptance, and making sense of past experiences (Lewis, 2016; Powell & Acker, 2016; Punshon et al., 2009). The sense-making process individuals engage in post-diagnosis has been framed as ‘biographical illumination’ (Tan, 2018) whereby individuals review their life history, facilitating a transformation of self-concept.

A large body of research describes the experiences of parents of children diagnosed with ASD. Common themes include a range of emotional reactions and appraisals (e.g., Lutz et al., 2012; Mansell & Morris, 2004; Midence & O’Neill, 1999; Mitchell & Holdt, 2014; Russell & Norwich, 2012). It is possible that parents of adults may experience similar emotional responses, changes to their identity, role and sense of self as parents of younger children. However, because of the large differences (in terms of rights and autonomy) between parenting an adult and a younger child and the longer time period to receive a diagnosis, it cannot be assumed that the impact will be similar.

Thus far, only one study has explored the experiences of parents of adult children recently diagnosed with ASD in adulthood (Raymond-Barker et al., 2016). Participants were six mothers of adults diagnosed with ASD in the preceding three years. Two over-arching themes and five sub-themes were developed. The first superordinate theme was ‘biographical continuity’ (Williams, 2000) which refers to the early stages of assessment being viewed as a continuation of the lifelong search for parent’s understanding of their adult child’s behaviour. The sub-themes concerned securing a referral and perceptions of the assessment process. The second superordinate theme was ‘biographical disruption’ (Bury, 1982) which described how parents re-examined their child’s and their own biographies to incorporate the diagnosis, and the frustration and disappointment that ensued when they attempted to mobilise their resources to adapt to the disrupted situation only to discover the lack of post-diagnostic support for themselves and their child. The sub-themes included the parents’ fight for post-diagnostic support for their adult child, their own support needs and fears for the future. Unlike the current study, this study focused solely on the assessment process rather than the longer-ranging impact for parents.

Lazarus and Folkman’s (1984) process model of stress and coping is suggested to account for individual differences in response to diagnosis of ASD, considering the role of appraisal of socio-ecological and personal resources in directing coping attempts (Punshon et al., 2009). Attribution theory (Weiner, 1985) is also relevant to parents’ process of making sense of their adult child’s behaviour following diagnosis as this new knowledge and understanding may result in changed attributions about causes and controllability, and affective and behavioural responses (Williams et al., 2012).

Many individuals with ASD continue to require support with numerous aspects of their daily lives including finding and keeping employment, making sense of social interactions, accessing higher education, and everyday living skills (Griffith et al., 2012), for which the majority is provided by their parents (Piven & Rabins, 2011). Parents of adults with ASD experience numerous burdens (Marsack-Topolewski & Church, 2019), often resulting in lower quality of life and reduced psychological wellbeing (Herrema et al., 2017). Part of parents’ adjustment to the ASD diagnosis may entail developing understanding that their adult child is likely to require lifelong support. NICE guidelines (2016) and the Think Autism strategy (Department of Health, 2014) recognise the potential impact on those supporting individuals diagnosed with ASD and advocate that carers should have an assessment of their own needs. However, in the reality of clinical practice, this is often not the case (Raymond-Barker et al., 2016).

Lack of post-diagnostic support is of similar concern to parents of adults (Raymond-Barker et al., 2016) as it is to parents of children (Crane et al., 2016) and individuals diagnosed themselves (Jones et al., 2014), resulting in feelings of frustration, disappointment, confusion and abandonment. The importance of post-diagnostic support for parents is highlighted by Crane et al. (2018), who recognise the potentially detrimental impact on parents of the discussion of difficult past experiences during assessment, and Punshon et al. (2009) who suggest that individuals and their families should have the opportunity to process the meaning of diagnosis to them with a professional.

Given the potentially detrimental impact on the psychological and emotional wellbeing of parents in light of their child’s diagnosis, it is important to understand their experiences and needs. Further understanding could inform the provision of support offered to parents regarding adapting to the diagnosis and effectively supporting their adult child. To extend on Raymond-Barker et al.’s (2016) findings, the current study will explore the wider ramifications for parents of the diagnosis rather than just the assessment process. The research questions the current study will qualitatively explore are:

1. What are the experiences of parents of adult children recently diagnosed with ASD in adulthood?
2. What are parent’s views on any informational or emotional support needs they have resulting from the recent diagnosis?

## Methods

A critical realist epistemological position was adopted in the present study. Critical realism assumes that knowledge of reality is mediated by beliefs and perceptions shaped by external factors, and stresses the importance of context in explanation and understanding (Maxwell & Mittapalli, 2007). From this position, it is acknowledged that the diagnosis of ASD is influenced by social, cultural, and historical factors; and that the meanings and experiences of parents are shaped by external factors. Researcher reflexivity is central to the critical realist position, and so the role the researchers’ experiences of working with adults and children with ASD and their families played was acknowledged in shaping the aims and interpretations of the study.

### Participants

Eleven parents (two fathers, nine mothers) from nine families participated. Seven participants were interviewed individually and four (Phil and Barbara, and George and Susan) were interviewed with their respective spouses. To be eligible to take part, participants were required to be a parent of an adult (aged 18 or above) diagnosed with ASD without intellectual disability within the three to six months prior to recruitment, be able to communicate verbally in English and provide informed consent.

All participants were biological parents, and the majority of their adult children were male (91%). Almost all participants identified as White British (82%) with two being from another ethnic group. Most were married (73%) whilst the others were divorced (18%) or widowed (9%). The adult children ranged in age from 20 to 37 years old. Most of the participants’ adult children lived independently (56%), whilst Jane, Mary, Rita and Karen’s^1^ adult children lived with them. Five participants were working either full- or part-time, five were retired, and one participant was unemployed. One participant disclosed that they also had a diagnosis of ASD themselves.

### Procedure

Ethical approval was obtained from the university research department, the local NHS Research and Development department, and the Health Research Authority.

Participants were recruited from a specialist ASD service which provides multidisciplinary diagnostic assessment for adults. An opportunity sample consisting of parents attending to participate in their adult child’s assessment was used. The intended sample size was based on recommendations in relevant literature (Clarke & Braun, 2015; Guest et al., 2006).

For the most part, the initial recruitment approach was made by the diagnosing clinician(s). Research posters were also displayed in the waiting room of this Service and an advert was placed on the Service’s social media site. During the diagnostic assessment, the purpose of the study was briefly introduced, potential participants were given an information sheet and were able to opt-in to be contacted. Those who opted in were contacted 10 weeks later to arrange an interview two weeks later. Participants were given the option of a face-to-face, telephone, or Skype interview to ensure equality of opportunity. All elected to be interviewed face-to-face.

An interview schedule of questions was collaboratively produced with clinicians experienced in diagnosing adults with ASD and with a parent with similar experience to that which this study was investigating, as well as the researchers’ reading of relevant literature to ensure all relevant topics were covered and questions were easily understood and acceptable. Interview topics included the impact of the diagnosis on parents’ understanding of themselves and their adult child, their relationships, and their support needs resulting from the diagnosis (see Appendix 1 for Interview Schedule).

All participants provided signed informed consent prior to being interviewed. They also completed a brief verbal demographic questionnaire to gain understanding of their personal contexts. The audio-recorded interviews ranged in length between 36 to 128 min (*M* = 77.50; SD = 30.44) and were transcribed verbatim.

### Analysis

An inductive-deductive, semantic level thematic analysis was conducted recursively following the guidelines provided by Braun and Clarke (2006, 2013). The method was chosen as it enabled the production of a rich, detailed analysis of the dataset as a whole, which is beneficial when exploring under-researched areas such as the topic concerned and was consistent with the chosen epistemological position. The inductive-deductive approach allowed for the data to first be read inductively and coded without preconceptions, then to be read through a lens of extant research in similar areas to guide coding. A semantic approach to coding was taken looking at the explicit, surface meanings of what participants said to describe their experiences in detail (Braun & Clarke, 2006). The analysis focused on producing a sufficient theoretical account (‘theoretical sufficiency’; Dey, 1999) rather than data saturation.

First, transcripts were read repeatedly, and initial ideas noted. Next, each transcript was coded line-by-line to capture everything of interest across the dataset, ensuring enough contextual information so that the codes could be understood when separated from the data. The text of each participant was assigned a different font colour to allow easy identification of the transcript from which the code was originally taken. A Word document was used to cluster codes together into groups of candidate themes. A thematic map was produced to visually represent the locations of candidate themes and the codes attached to these in relation to each other. This provisional thematic map was reviewed in research supervision allowing some themes to be collapsed into each other and refined, and codes to be redistributed accordingly. It was then considered how these codes fitted together to form overarching themes and subthemes with all relevant data collated. Provisional themes were discussed within the research team until agreement was reached. Next, the coherence of each theme and the ability of the themes to reflect the dataset as a whole was considered, and a final thematic map was produced to show how the themes fitted together. Themes were then refined and named, and illustrative extracts were chosen. Extant literature was used to contextualise the analysis, highlight continuities and differences, and extend the insights gained from the current dataset. A reflexive diary was kept by the first author to maintain awareness of assumptions and biases. Initial coding of the first transcript was checked by the second author and the analysis was discussed extensively in supervision. Quality of analysis was monitored by the first author using checklists by Braun and Clarke (2006) and Elliott et al. (1999).

## Results

### Overview of Themes

**Fig. 1:**
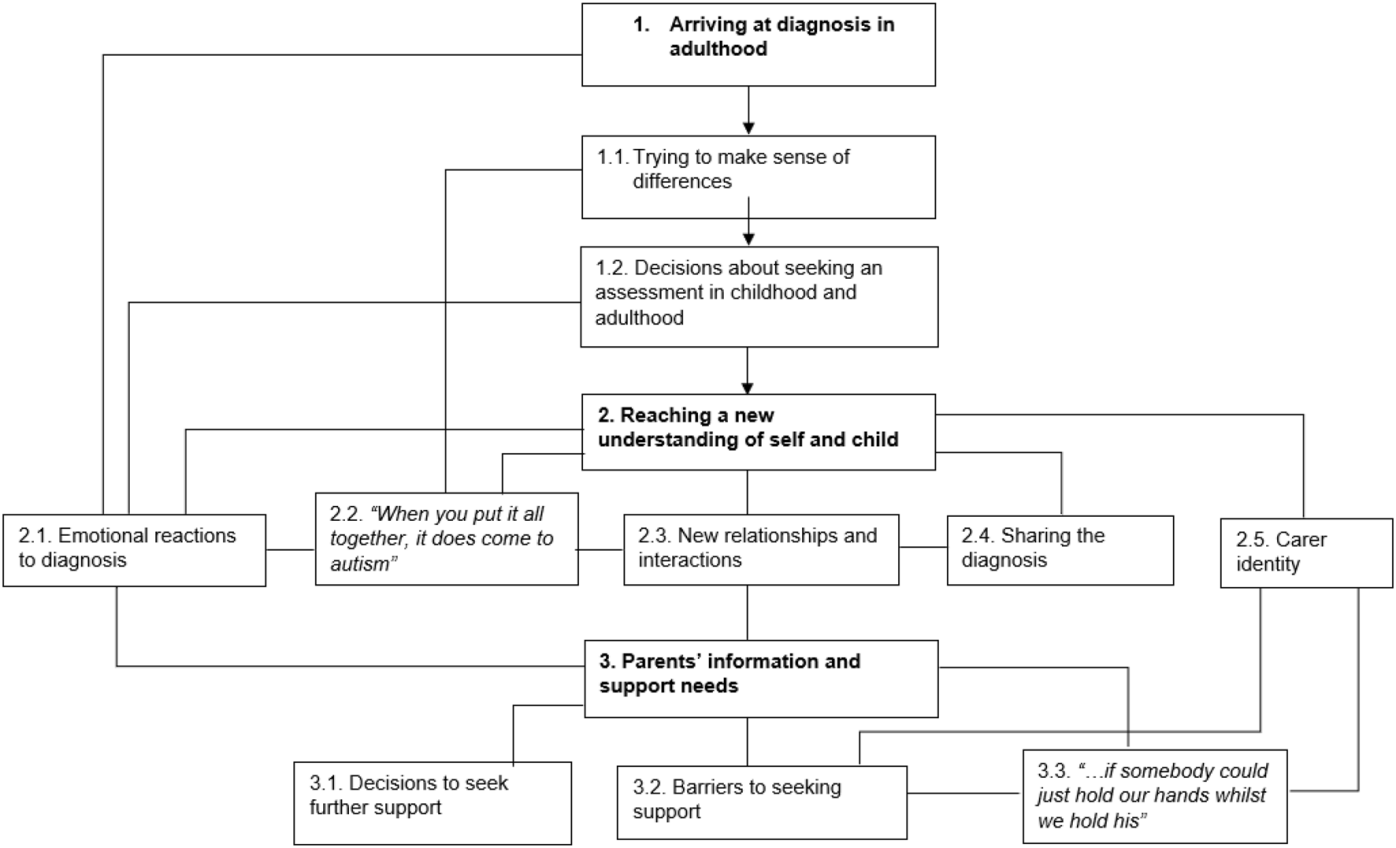
Thematic map

Three overarching themes with related subthemes were identified and are summarised in Figure 1. Each theme and subtheme is distinct, but connecting lines indicate represent connections made between themes and subthemes in the process of analysis. The central theme of ‘Reaching a new understanding of self and child’ will be described in depth as this is most important for understanding the psychological impact on parents with a briefer overview of the other themes. Illustrative quotes are offered throughout.

## Arriving at Diagnosis in Adulthood

### Trying to Make Sense of Differences

The majority of parents had recognised that their child had difficulties from school age but “didn’t know why”. This led parents to generate various attributions for their behaviour including being “a bit backward”, “fussy”, “shy”, “stubborn”, “awkward”, or “naughty”.

### Decisions About Seeking an Assessment in Childhood and Adulthood

Parents who considered their child’s differences to have an underlying pathology (for example as opposed to attributing them to aforementioned characteristics such as fussiness or shyness) chose to pursue assessment. However, they were met with reluctance, blaming and dismissive responses from professionals:

No matter who I went to see, the doctor always said it was me. It was me they decided to put on anti-depressants, and I was going for help for [my son], but all the time it was “I’m spoiling him”, “I’m wrapping him in cotton-wool”. (Jane)

Some parents whose child’s differences were highlighted by professionals chose not to pursue assessment due to concerns about their child being “excluded” from mainstream education, subjected to “stigma” and the label damaging their self-esteem. Karen, having witnessed her son’s negative reaction to his dyslexia diagnosis previously believed he would have “fought” an ASD diagnosis and wanted to protect his sense of identity:

When you’re an adolescent you don’t want to be different, you want to be the same…. So, you get a diagnosis, you’re not the same as your friends.

Later in life, some parents’ adult children’s difficulties were highlighted by professionals involved in managing their mental health needs, whilst other parents were more instrumental in the referral process, raising the possibility of seeking assessment themselves in a continued attempt to make sense of their child:

It was me that said to him last year… “why don’t you go and get yourself sorted?...Go to your doctors and ask to be referred ‘cos there’s something wrong chemically, maybe an imbalance”. (Susan)

For parents who had taken an anti-diagnosis position during childhood, re-appraisal of attributions for their child’s behaviour and their own coping resources prompted the decision to seek assessment. Karen recognised that she was less able to meet her son’s needs in adulthood and could benefit from support which could only be accessed via diagnosis:

As an adult, I can’t support him to socialise, I can’t go out with him on dates. I can’t go with him to the pub or the club.

## Reaching a New Understanding of Self and Child

### Emotional Reactions to Diagnosis

A range of emotional reactions to the diagnosis were expressed, reflecting the complexity of the experience. The expectation that a diagnosis would be made resulted in “relief” and “satisfaction” for some parents when the diagnosis was received. However, other parents described feeling incompetent for not having recognised the possibility of ASD:

I felt a bit useless, not realising that that (ASD) was the matter. (Mary).

Some parents felt “big guilt” due to beliefs that they had “let their child down” (George) or “failed” them by not pursuing childhood assessment further, or for their previous negative attributions for their child’s behaviour and management strategies employed:

…I did go away feeling very guilty that I hadn’t tackled it over the years…it brought back lots of thinking about that and how we did deal with her and how we did cope, which made me think, yes, you know, perhaps there were lots of things I could have done differently. (Kate)

### “When You Put it All Together, It Does Come to Autism”

The majority of parents could be considered to be experiencing ‘biographical illumination’ with the diagnosis being used as a lens through which to make sense of their child explaining sensory sensitivities, “obsessions”, difficulties with relationships, differences in communication and “meltdowns”, which had powerful emotional consequences for some. For example, Linda was able to attribute a different meaning to her son’s response to her crying when leaving him at university for the first time, and to reflect on this humorously rather than with sadness:

I ended up in floods of tears and he just looked, I don’t know, he just looked really bewildered, you know, at why I was crying really. You know, “I’ve got to go now mum,” and he disappeared up the road. And he looked round as if to, you know, “what the hell’s the matter with you?” We laugh about that to this day, hilarious really, when you think about it. (Linda)

However, some parents could be seen as experiencing ‘biographical disruption’, ruminating on their past actions and decisions and having a significant and enduring impact on their self-perception:

…it’s made me feel more of a failure in the sense of, “maybe I should have done this”. So yeah, it made me not be confident in myself. It’s made me re-look at myself…it’s made a difference to how I feel and I’m still trying to understand it. I’m not there yet”. (Tracey)

### New Relationships and Interactions

Knowledge and understanding of the diagnosis allowed almost half of the parents to reconsider their attributions, changing their behaviour and emotional reactions. For example, Karen and Jane discussed how the diagnosis had allowed them to be more objective and not “get so frustrated and hold on to things”:

So, what I would do previously, he’d be rude and then I’d say “no, I can’t talk to you, you’ve been too rude”. And then, obviously, that just makes him anxious…. so, I don’t do that now. I’ll just say, OK, he’s back to normal, that’s fine. (Karen)

Some parents expressed how their child’s diagnosis had allowed them to feel “definitely closer” to their adult child, enabling understanding of past ruptures in their relationship, new attributions, and forgiveness:

…they’ve suddenly turned into a father and a son, which they’ve never been, not since [son] was about two or three, since he stopped reading stories at night. They had that close bond then, suddenly that close bond is back. (Jane)

Other parents seemed unsure whether to and how to change when considering their sons’ diagnoses. Linda felt a greater need to protect and care for her son, possibly reflecting her concern about stigma, but felt she had to fight the urge not to “smother him”, be “overly clingy” or “too clucky”:

I don’t know, I just feel, now that he’s got this, you know, label, I just feel maybe I should look out for him more than I would my other son, whether that’s right or not.

Tracey experienced conflict as she had some doubt about her son’s diagnosis believing him to be “capable” of learning and understanding that “there are certain things that aren’t acceptable”, but also wondered whether she was unfairly overestimating his abilities:

Am I being harsh in my expectations and it’s that he can’t help it? Is it that I am misjudging things?...I don’t want to enable him and excuse certain things. But am I right? Am I wrong? I don’t know. (Tracey)

Several of the parents discussed how their adult child’s diagnosis had led to positive changes in their relationships with their spouses. Barbara described how she used to “fall out” with her partner over his handling of situations with their son creating “great animosity” between them. Since the diagnosis, Phil’s changed attributions towards his son have seemingly allowed him to forgive his past behaviour, enabling him to respond with more patience and empathy:

We can say 100% now, it’s not [son’s] fault. There was always a slight doubt in the past, “is he just being awkward with me?” So, to have it confirmed that he’s got this condition and the way he’s behaved is not his fault made me feel a lot better, knowing he wasn’t just picking on me to be nasty.

Jane and Kate’s husbands’ improved understanding of their children encouraged them to take a more active role in caring for and supporting them, allowing the mothers to discuss their concerns more openly, resulting in a sense that they were no longer “bearing all the burden”:

It’s a relief! It takes something off of me. I’m not the only one looking out for [son]. I leave for work at 8 am and I know my husband will get [son] up and he will get to work…I know it’s not just me running around after him. (Jane)

The diagnosis had a limited impact on behaviour, communication and relationships for several parents. For George and Mary, it seemed that this response was determined by their adult child’s relatively minimal need for care and support from them, whilst for Rita and Barbara, it was their perception that they already had a good understanding of their sons’ needs and were supporting them adequately.

### Sharing the Diagnosis

Parents had different motivations for sharing the diagnosis with family and friends, the most common being to correct or avoid negative attributions for their adult child’s behaviour. Several parents mentioned that the diagnosis had given them a more acceptable way of explaining their child’s behaviour in social situations where they may be perceived as being “awkward” or “rude”.

Some parents shared the diagnosis with family in the hope that it would encourage empathy for their child as well as garnering social support, but this did not always come to fruition, leading to disappointment and frustration:

They look at him as if he’s done something or said something and I get angry with them for not understanding. (Barbara)

Other parents appeared not to have shared the diagnosis as extensively, perhaps reflecting the more minimal impact it had had on their understanding of their child. Mary’s son’s diagnosis of ASD may have been less significant to her than her own diagnosis, possibly influencing her lack of desire to discuss it with friends and family:

I suppose it’s something you don’t need to talk about all the time. Unless I’m asked a question about it, I’ll answer it. I suppose if you need to talk about it, you will. (Mary)

### Carer Identity

Given their continued high involvement in their children’s lives, the diagnosis served to reaffirm their already established carer identity for the majority of the sample. Only Karen’s perception of her role changed when a professional referred to her in this way:

Well, when he (son) had to see a clinical nurse specialist the first time, and the nurse said to me, “you are his carer”. He said, “no, you are”.

Prior to this point, Karen had not thought of herself in this way, but reflecting on what the nurse had said lead her to reconsider her identity in relation to her son.

Parents whose adult children had less significant needs tended not to ascribe to the carer label with Linda describing what she did for her son as “sort of a support role, I suppose”.

## Parents’ Information and Support Needs

### Decisions to Seek Further Support

Parents’ employment experiences, existing knowledge and access to social support, and their child’s care and support requirements determined their support needs. Some parents were disappointed and frustrated by the lack of post-diagnostic support and there was a sense of abandonment:

It was a bit like, “[son] has got Asperger’s. Thank you very much. Goodbye.” and that’s the end of it…they were lovely people, but they can’t offer us what’s not there. (Phil)

The lack of support had significant psychological consequences for some, impacting on their sense of parental self-efficacy. Tracey described feeling “lost”, “blind” and “in the dark” following her son’s diagnosis and de-skilled in her ability to support him:

It’s just very difficult knowing what to do, what not to do. When he was younger, I thought I did the right thing, but now he’s older, I don’t know what to do. Will I reflect again and think, “I should have done this?” You don’t want to make the same mistake twice.

### Barriers to Seeking Support

Several parents described their difficulties in accessing relevant, easily digestible, good quality information about ASD, leading some to feel overwhelmed.

I mean obviously you can look online, but you know, if you look up online, you get ten different completely conflicting things about it, won’t you?... (Linda)

Others discussed the obstacles they had encountered in trying to secure support for themselves and their children including a lack of information about high-functioning individuals and an absence of support groups for parents of individuals diagnosed in adulthood.

### “If Somebody Could Just Hold Our Hands Whilst We Hold His”

The most significant need expressed by over half of the parents was for emotional and relational support. Some parents stated this could be best met through contact with other parents of late-diagnosed children to allow normalisation and sharing of coping strategies:

If it was just a group where there was other mums and dads that have got high functional late diagnosis where you can sit and laugh about some of your situations, but you know you’re not the only one that’s got that…if you’d had that experience, you could talk through how you’d got through that. (Jane)

Conversely, other parents stated a preference for “expert” involvement to support them in understanding their child’s diagnosis and needs, perhaps reflecting uncertainty about how to support their children and doubts about their parenting abilities:

Someone to ask, “OK, he’s got this diagnosis. What does that mean now? Do I need to do anything? How is it manifesting in him? How’s it impacting?” There’s nothing there and I’m trying to just figure it out. (Tracey)

Given their unmet support needs, some parents were concerned about their children’s futures with those with most involvement having the greatest concerns. This “worry” was brought into sharp relief for Rita, whose husband had died suddenly:

I mean I’m seventy-eight, I’m not going to last forever. Obviously, my husband was eighty. That was a shock, you know, he was always fit and healthy….It brings it home more now that he’s died that, you know, it could happen to me any day sort of. And what will happen to [son]?

Several parents expressed a desire for more information than that provided by the service, such as “proper guidance” about where to access reliable information about ASD and signposting to services concerned with benefits and supported living accommodation.

## Discussion

This was the second study to explore parents’ experiences of their adult child receiving a diagnosis of ASD in adulthood. The present study differed from the first by Raymond-Barker et al. (2016) exploring the wider ramifications for parents of the diagnosis rather than just the assessment process.

As in Raymond-Barker et al.’s (2016) study, the majority of parents had recognised differences during childhood, and some who chose to pursue diagnosis experienced unhelpful or even negative responses from healthcare professionals. This has important implications about the development of knowledge of healthcare professionals working with both children and adults in recognising ASD symptomology and having an awareness of local referral pathways to allow for earlier diagnosis and access to support.

Some parents described having rejected the possibility of seeking diagnosis due to their concerns about stigmatisation or exclusion of their children. Advocates of the neurodiversity movement purport that viewing ASD as a different but positive and equal way of being may reduce perceptions of stigma and encourage parents to seek diagnosis (Kapp et al., 2013).

Parents’ accounts demonstrated that they experienced broadly similar emotional reactions as parents of children (e.g., Lutz et al., 2012; Mansell & Morris, 2004; Midence & O’Neill, 1999; Mitchell & Holdt, 2014; Russell & Norwich, 2012) and adults diagnosed themselves (Jones et al., 2014; Powell & Acker, 2016; Punshon et al., 2009; Tan, 2018). However, although research with parents of children suggests that some experience guilt (e.g., Midence & O’Neill, 1999), this appeared to be a more prominent emotional experience in the present study related to not having recognised the possibility of ASD, not having pursued childhood assessment more thoroughly, parents’ previous negative attributions for their child’s behaviour and the ways they responded to this.

Extending Tan’s (2018) suggestion that ASD diagnosis can engender ‘biographical illumination’ for those receiving a diagnosis in adulthood, the majority of the parents in this study engaged in a review of their child’s life history using their new knowledge of ASD to make sense of their adult child’s sensory sensitivities, “obsessions”, difficulties with relationships and communication. It also encouraged them to re-evaluate their prior attributions about the cause and controllability of their child’s behaviour allowing them to forgive and reconcile difficult past interactions with their child, develop patience, empathy and tolerance, respond more sensitively to their child’s communication, and build closer relationships. For some couples, having developed a shared understanding of their child had a positive impact on their marital relationships and resulted in a reduced sense of burden for mothers. Some parents shared their adult child’s diagnosis with their wider network in the hope that they would experience the same ‘biographical illumination’ that they had, becoming frustrated when it did not have the impact they had hoped. However, as in Raymond-Barker et al.’s (2016) study, some parents also experienced ‘biographical disruption’ when they considered that they had made poor decisions and not supported their child adequately, having a detrimental impact on their self-esteem. The diagnosis also left some parents feeling de-skilled and unsure how best to support their child, perhaps relating to their appraisal of the diagnosis as threatening and their resources for coping with it as lacking (Lazarus & Folkman, 1984).

Supporting existing literature, the majority of parents in this study had significant involvement in supporting their adult children in their daily lives, even when they were not living with their children (Griffith et al., 2012; Piven & Rabins, 2011). The diagnosis therefore served to reaffirm their status as carers for the majority, although this depended on the needs of the child.

Corroborating previous findings (Crane et al., 2016; Raymond-Barker et al., 2016), parents in the present study expressed their desire for emotional and relational support either through contact with professionals or peers, and guidance about where to access reliable information and relevant support services. They also expressed frustration, disappointment and abandonment regarding the lack of post-diagnostic support. Crane et al. (2018) and Punshon et al. (2009) advocate that parents should be given the opportunity to talk with a knowledgeable professional about what the diagnosis means for their family. Also, NICE (2016) guidelines state that families and carers of adults with ASD should be offered an assessment of their own support needs, be given advice on obtaining practical support and in planning the future care of the diagnosed individual. However, the current findings would suggest that this is not happening in routine clinical practice, possibly due to demand exceeding available resources. Given the reported unmet needs of parents and the potential impact this may have on their ability to support their adult child, this is an important area to be addressed. Potential targets for post-diagnostic support suggested by the findings would be around boosting parental self-efficacy through developing understanding of how ASD affects their adult child and building coping resources.

The findings should be considered by clinicians involved in the diagnosis of ASD in adults to allow them to prepare parents for their possible emotional reactions, the potential changes, both positive and negative, that they and their families may experience in terms of self-perception and relationships, and to encourage them to consider how they may cope with these issues. Acknowledging financial pressures on services, it is necessary that clinicians are open from the outset about the lack of support for adults with ASD and their carers to encourage realistic expectations about the outcome of diagnosis. However, given the continual high levels of support a lot of the parents provided to their children and their expressed unmet needs, it is paramount that services provide good quality information about ASD including useful websites to access, and signposting to relevant local support groups and agencies who can advise on issues such as accommodation and benefits. It is also important to recognise the complexity and diversity of the experience and to tailor post-diagnostic support according to the level of need of the parent and child. The findings may also be useful to parents whose adult child has recently been diagnosed to allow normalisation of feelings and possibly provide hope and encouragement.

## Limitations

It is of course important to acknowledge the limitations of the study. As the sample was self-selected, it likely reflects the fact that these parents continued to have significant involvement in their adult children’s lives, and so the findings perhaps would not reflect the experiences of parents who are less involved. Specific information was not gathered regarding whether the adult children lived with their parent(s) or not, but it may be useful for future research to explore whether there are notable differences between parents with their adult children living at home and those who lived separately. The sample lacked diversity as there were only two fathers, one parent of a daughter, and two participants from ethnic backgrounds other than White British. It is important that future research attempts to recruit more diverse samples to understand any differences in experiences and needs of these groups.

Retrospective qualitative studies based on participants’ recall have methodological limitations, which could be overcome by longitudinal and prospective designs beyond the scope of the present study. This would allow data to be gathered at different timepoints, through the process of seeking a diagnosis and after one was given. As most parents were interviewed individually and diagnosed adult children were not interviewed at all, there is no verification of the spouses’ responses or the children’s perceptions of the changes in their parents. Future research could interview dyads composed of parent and child or mother and father to seek this additional understanding. Gaining the perspectives of siblings of diagnosed individuals would also help in developing an inclusive understanding of the whole family's lived experience and support needs, as well as offering an opportunity to triangulate the data.

## Implications for Future Research

Although self-efficacy was not measured in this study, it appears that for at least some parents their adult child’s ASD diagnosis led to a decrease in parenting self-efficacy. Future research could use formal assessments to establish whether the diagnosis does in fact impact on parenting self-efficacy, then whether interventions designed to bolster self-efficacy can achieve positive outcomes.

Given that parents highlighted the need for online support forums, informational resources, and post-diagnostic support groups, future research could concentrate on the development of these through focus groups with parents of adults with ASD to promote co-production and co-design of materials. These would then need to be piloted and evaluated to see whether they have any effect on parenting-related outcomes such as self-efficacy and stress, as well as seeking qualitative feedback.

## Conclusion

Findings demonstrate that parents experience complex emotional reactions to diagnosis, with guilt being predominant for many, but that the new knowledge of diagnosis can facilitate changes in understanding, attributions, interactions and relationships. However, the lack of post-diagnostic support can result in unmet support needs. These findings should be considered by clinicians involved in adult diagnosis of ASD to guide conversations about expectations and potential consequences of diagnosis, as well as informing the provision of post-diagnostic support.

## Appendix 1

- 1. What support (if any) do you currently provide your child?
- 2. Did you ever seek an assessment for your child as they were growing up or is this their first time of seeking a diagnosis? (either for autism or other developmental conditions).

Prompt: If yes, what conditions? What was the outcome?

If it hadn’t been considered before, where you surprised when they said they were going to be assessed?

- 3. What was your understanding of autism before your child received this diagnosis?
- Prompts: Had you heard of it?
- 4. When you came to the diagnostic assessment, what were your expectations of diagnosis for you?
  - a. Did you expect it to change the way you think of yourself or your child?
  - b. Did you expect to have any emotional reactions?
  - c. Did you think it would make any difference to your relationship with your child?
  - d. Did you think it would make any difference to your family?
- 5. How has your understanding of autism changed since they received the diagnosis?
- 6. Does the diagnosis of autism make sense to you for your child?

Prompt: Is there another label you feel is more appropriate?

- 7. Has the diagnosis had any impact on how you see or understand your child? If so, what?
- 8. Has the diagnosis had any impact on your relationship with your child? If so, what?

Prompts: Do you feel closer? Further apart?

- 9. Has your child’s diagnosis made any difference to how you see yourself?

Prompts: As a parent? As a person?

- 10. Do you consider yourself to be a carer for your child?

Prompts: If so, how do you feel about being a carer for a person with autism?

- 11. What support needs do you have as a carer for your child?
  - a. Did or do you have any emotional support needs?
  - b. Did or do you have any needs accepting and adjusting to the diagnosis?
  - c. Did or do your family have any emotional support needs?
  - d. Did or do your family have any needs accepting and adjusting to the diagnosis?

## Footnotes

1 Pseudonyms for both parents and their adult children have been used throughout to protect their anonymity.
